# Systolic third sound associated with systolic anterior motion of the mitral valve in cats with obstructive hypertrophic cardiomyopathy

**DOI:** 10.1111/jvim.16806

**Published:** 2023-07-13

**Authors:** Vittorio Saponaro, Clémence Mey, Irène Vonfeld, Antoine Chamagne, Maria‐Paz Alvarado, Jean‐luc Cadoré, Valérie Chetboul, Loïc Desquilbet

**Affiliations:** ^1^ École Nationale Vétérinaire d'Alfort Maisons‐Alfort France; ^2^ VetAgro Sup Marcy‐l'Etoile France; ^3^ Université Paris Est Créteil, INSERM, IMRB Créteil France

**Keywords:** agreement with echocardiography, feline, new auscultation findings, phonocardiography

## Abstract

**Background:**

Third heart sounds in cats frequently are associated with hypertrophic cardiomyopathy (HCM) but their exact characterization and timing within the cardiac cycle remains unknown.

**Objectives:**

Characterize third heart sounds in cats by phonocardiography and test the ability of 3 observers with different levels of experience and training to recognize third systolic heart sounds in cats.

**Animals:**

Fifty client‐owned cats of different breeds presented for heart screening.

**Methods:**

Cats were prospectively assessed using an electronic stethoscope (with digital recording) and then underwent full conventional echocardiographic examination. Audio recordings were blindly assessed in a random order by 3 observers: the cardiologist who collected clinical data, as well as a trained and an untrained junior veterinarian. Cohen's kappa coefficients were calculated to quantify agreement between the opinion of each observer and the echocardiography results (considered the gold standard).

**Results:**

Twenty cats had a third systolic sound on phonocardiography and an obstructive HCM phenotype with systolic anterior motion of the mitral valve (SAM) on echocardiography. Agreement with echocardiography was very good for the experienced cardiologist, substantial for the trained junior veterinarian, and poor for the untrained junior veterinarian (kappa of 0.92, 0,64, and 0.08, respectively).

**Conclusions and Clinical Importance:**

We describe here a new auscultatory abnormality in cats with obstructive HCM. It could help a trained non‐cardiologist veterinarian in suspecting obstructive HCM in cats based on auscultation only.

**Abbreviations**
HCM: hypertrophic cardiomyopathy
LVOT: left ventricular outflow tract
PCG: phonocardiography
SAM: systolic anterior motion of the mitral valve
SAM‐AS: SAM‐associated sound

## 1 INTRODUCTION

In a healthy individual cat, under normal physiological conditions, the audible sounds on heart auscultation are S1 and S2, which correspond to closure of atrioventricular valves and of sigmoid valves, respectively. Both heart sounds define the beginning and the end of systole. Low‐frequency diastolic heart sounds, also known as gallop sounds, can be heard in specific physiopathological conditions such as left ventricular volume overload and atrial forced contraction. These settings can be characterized respectively by an early‐diastolic sound called S3 and a late‐diastolic sound called S4.^1^ Gallop sounds have been associated commonly with hypertrophic cardiomyopathy (HCM) in cats, which is the most common heart disease in cats, with a prevalence of approximately 15% in the general cat population.^2^ Gallop sounds have been reported in 2.6% to 8.7% of cats with subclinical HCM,^2^, ^3^ making this finding useful to establish an early diagnosis. However, gallop sounds in HCM cats have not been specifically studied by phonocardiography (PCG),^4^ and recent consensus on cardiomyopathy in cats defines gallop sounds using the more appropriate term “third sounds” because their characterization and timing within the cardiac cycle remains unclear.^2^, ^5^ Therefore, the existence of specific third systolic sounds in HCM currently has not been ruled out. Our hypothesis was that third sounds in cats with HCM also could occur in systole. Our study therefore had 2 main objectives: (a) characterize third heart sounds using PCG in cats diagnosed with HCM and (b) test the ability of observers with different levels of experience and training to recognize third systolic sounds in cats diagnosed with HCM.

## 2 MATERIALS AND METHODS

**FIGURE 1:**
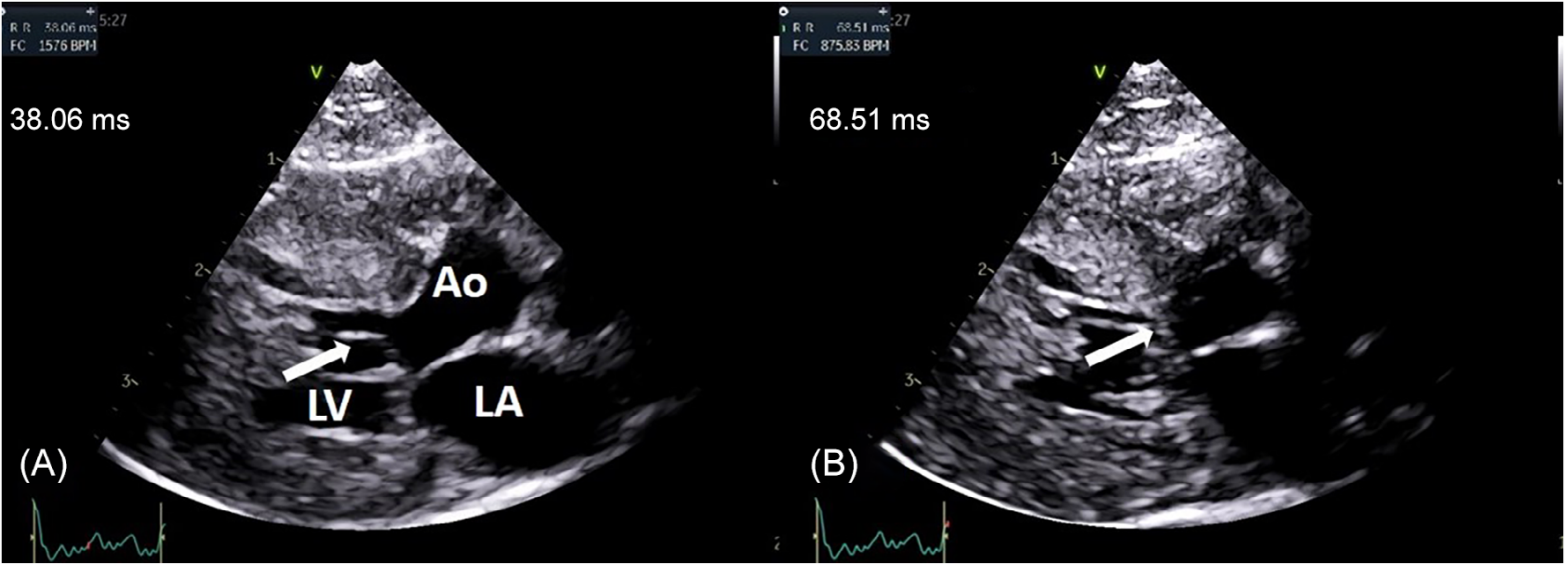
(A,B) Right parasternal long axis 5‐chamber view, same cat of Figures 2B and 2C. The systolic anterior motion of the mitral valve (white arrow) is directly visualized. Time to SAM (displayed in milliseconds) is measured by the aid of concomitant ECG, between the R wave and the ECG cursor fixed at the first frame in which the mitral anterior leaflet touches the interventricular septum (B). Ao, aorta; LA, left atrium; LV, left ventricle.

**FIGURE 2:**
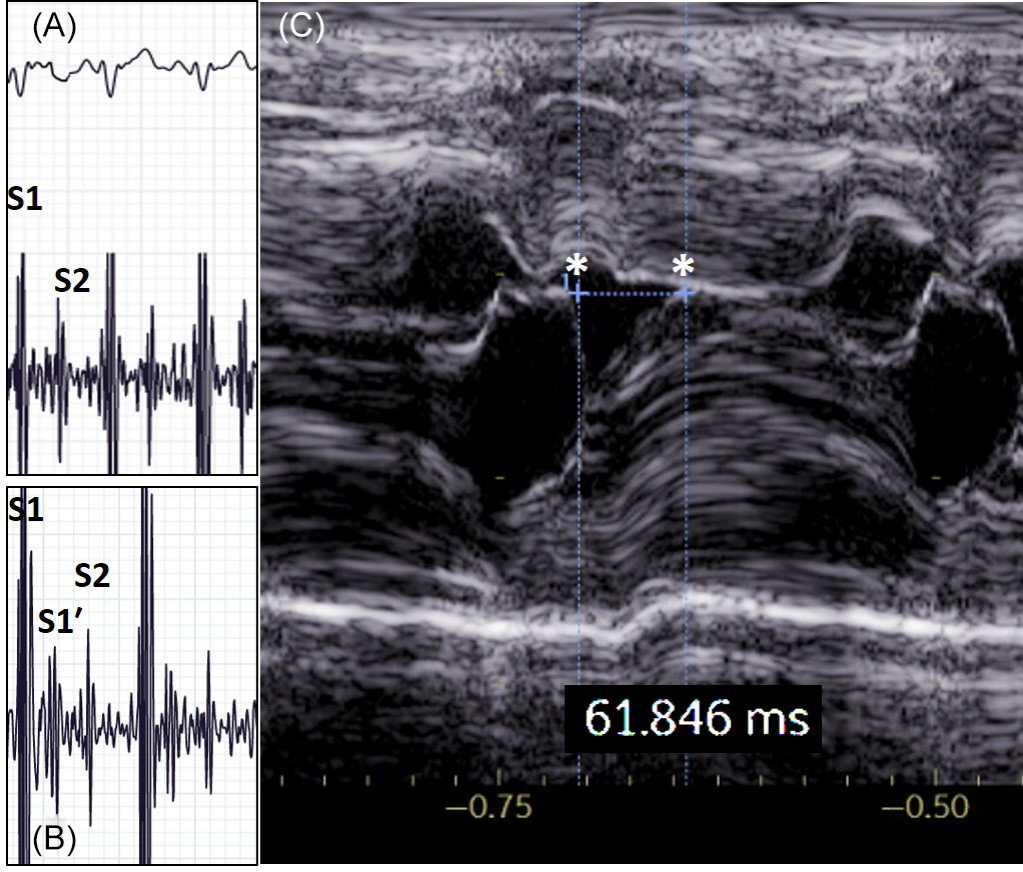
(A) Phonocardiogram of a normal cat with simultaneous ECG. Paper speed: 25 mm/s, ECG amplitude: 10 mm/mV. S1 is simultaneous to QRS complex and taller than S2 which coincides with the end of T wave. (B) Phonocardiogram of the same cat of Figures 1 and 2C, showing a hypertrophic cardiomyopathy phenotype with systolic anterior motion of mitral valve. Notice the additional systolic signal (S1′) after S1 and before S2. It appears smaller than S1 and as tall as S2. It is separated from S1 and occurs almost 70 ms after S1. Paper speed: 25 mm/s. (C) M‐mode echocardiographic appearance of the systolic anterior motion of the mitral valve in the same cat of Figures 1 and 2B. Notice the 2 white asterisks marking the beginning of the systole and occurrence of SAM (the beginning of the collision of the mitral anterior leaflet with the interventricular septum), the interval is displayed in milliseconds. Notice the correspondence between the timing of appearance of S1′ on phonocardiography in Figure 2B, and time to SAM in Figure 2C, in the same cat.

All cats referred for the first time to the cardiology department of Alfort National Veterinary School, between October 2020 and February 2021 and having a complete cardiac scan by the same operator (Vittorio Saponaro), were enrolled. Cats were referred for cardiac screening, investigation of a heart murmur, assessment because of heart failure, or for complete preanesthetic assessment. Auscultation was performed by an experienced veterinary cardiologist with 20 years of experience (Vittorio Saponaro, observer A) first using a conventional stethoscope (3 M Littmann Master Cardiology) and then using an electronic stethoscope (3 M Littmann CORE Digital Stethoscope) on both sides of the chest wall at the apex first and then at the base, in a quiet room with simultaneous PCG recording. In some cases, electronic auscultation using PCG was performed with simultaneous ECG (Eko DUO ECG + Digital Stethoscope). Acquisition of PCG was conducted using the factory preset for cardiac auscultation: filters of 45 to 750 Hz or 100 to 500 Hz, duration of 15 seconds and paper speed of 25 mm/s. Phonocardiography recording was repeated at least 3 times starting from when observer A was satisfied by the auscultation performance. During the recording phase, another clinician (Clémence Mey), unaware of the aim of the study and not directly involved in the test of agreement and other data collection, stored the recordings on the computer [Correction added after first online publication on 4 August 2023. Clémence May has been corrected to Clémence Mey.]. No post‐processing was done on the original files and they were identified only by a code number corresponding to the cat and the date of the recording. Echocardiography (Vivid 7, Vivid E9; General Electric Medical System, Waukesha, WI equipped with 12 and 6 MHz probes) was performed on awake cats, gently restrained in standing position or in lateral recumbency, by the same trained operator (Vittorio Saponaro). Echocardiographic variables were obtained from standard 2‐dimensional and M‐mode images and by conventional Doppler mode, acquired from the right parasternal short‐axis and long‐axis views and the left apical view. The right parasternal short‐axis view was used to record the left ventricular M‐mode variables including left ventricular internal dimensions and wall thicknesses at end‐systole and end‐diastole, fractional shortening, left atrial‐to aortic‐ratio and right ventricular outflow tract Doppler peak flow velocity. The right parasternal long axis view was used to determine the presence or absence of systolic anterior motion of the mitral valve (SAM) and, when concomitant ECG was present, the time to SAM was measured as the interval (expressed in milliseconds), between the R wave and the ECG cursor fixed at the first frame in which the anterior mitral leaflet touched the interventricular septum (Figure 1B). From the same view, time to SAM was also obtained in M‐mode as the interval between mitral valve closure and the beginning of the collision of the anterior mitral leaflet with the interventricular septum (Figure 2C). Left ventricular outflow tract (LVOT) peak flow velocity was interrogated by continuous wave Doppler in the left apical 5‐chamber view, guided by color Doppler. Measurements were averaged over 3 consecutive cardiac cycles except for right and LVOT peak flow velocities for which the maximal values were collected. The different cardiomyopathy phenotypes were diagnosed according to the consensus guidelines for echocardiographic classification.^5^ In particular, nonobstructive HCM was diagnosed if end‐diastolic left ventricular wall thickness was ≧6 mm measured using 2‐dimensional and M‐mode echocardiography as previously described,^3^ and end‐diastolic left ventricular internal dimension being in the reference intervals of published allometric scaling.^6^ Obstructive HCM with SAM was diagnosed if the previous criteria were met and if the septal leaflet of the mitral valve was displaced towards the septum in systole, obstructing the LVOT, ascertained by the following criteria: concomitance of color Doppler systolic aliasing through the mitral valve and through the LVOT; LVOT spectral Doppler‐derived peak flow velocity >2.5 m/s and a dynamic obstruction profile (mid and late systolic acceleration); and 2‐dimensional and M‐mode visualization of SAM.^5^ Restrictive phenotype was diagnosed in the event of normal left ventricular dimensions (including wall thickness) with left atrial or biatrial enlargement, with or without the presence of prominent bridges between the interventricular septum and left ventricular free wall.^5^ Echocardiography and PCG were reviewed in a blinded fashion at the end of the recruitment period.

Phonocardiographic tracings (Figure 2A,B) were analyzed only if of good quality (ie, proper baseline without background noise). Tracings that were interrupted by extracardiac artifacts (eg, tremor, purring or vocalization) were analyzed in the proper segments. Cats with inadequate PCG tracings were excluded from the study. At least 3 consecutive cardiac cycles were evaluated. When concomitant ECG was lacking, S1 was identified as the taller (amplitude) and longer (duration) PCG signal of the 2 main sounds, based on experience and the veterinary and human medical literature.^7^, ^8^ When a third PCG signal was present it was classified as systolic (S1′, to distinguish it from S1, the other nearest sound), early‐diastolic (S3) or late‐diastolic (S4). When S1′ was present, the interval in milliseconds from S1 was measured. For the test of agreement, at least 3 digital audio recordings of cardiac auscultation from each cat were collected. The audio files were grouped for each cat and randomly ordered and played back for off‐line auscultation on different occasions by 3 veterinarians with different levels of experience (observers A, B, and C). At the time of test of agreement, observers were blinded to signalment, medical history, PCG and echocardiographic findings. Observer B (Irène Vonfeld) was a cardiology intern (2 years since graduation) who received brief training from observer A in cardiac auscultation and detection of third sounds in cats, before participation in the study. Observer C (Antoine Chamagne) was a rotating intern (1 year since graduation), untrained in cardiac auscultation and detection of third sounds (specifically in cats). To insure homogeneity of listening conditions, the 3 observers wore the same headphones and listened to the original audio files without any change in the manufacturer's settings. They were asked if they detected a third systolic sound or not, independently from other abnormal auscultatory findings, such as murmurs. Moreover, observer A qualified the third systolic sound as loud or soft.

### 2.1 Statistical analysis

Agreement between the presence (vs absence) of obstructive HCM with SAM on echocardiography (considered the gold standard) and auscultatory detection of the presence (vs absence) of a third systolic sound on digital auscultation was quantified by calculating Cohen's kappa.^9^, ^10^ Kappa values <0.20 indicated poor agreement, between 0.21 and 0.40 indicated fair agreement, between 0.41 and 0.60 indicated moderate agreement, between 0.61 and 0.80 indicated good agreement, and between 0.81 and 1.00 indicated very good agreement.^9^, ^10^ The qualitative subjective description provided by observer A was not taken into account in the binary statistics (presence/absence) of the test of agreement with echocardiography.

For descriptive purpose only, the median and interquartile range (IQR) of LVOT gradients were calculated separately for cats presenting with a loud sound and for those presenting with a soft or intermittent sound, according to the qualitative description of observer A. The Mann‐Whitney test was used to compare the 2 medians. Alpha type‐I error was set at 0.05.

## 3 RESULTS

### 3.1 Clinical and instrumental data

Fifty‐four cats were enrolled in the study but 4 were excluded because of poor quality PCG recordings. Fifty cats finally were included, 27 males and 23 females. Breeds were represented as follows: domestic shorthair (44), Maine Coon, Norwegian (2), Siamese (1), Persian (1), and Birman (1). Median (IQR) age was 3.1 (1.1‐10.8) years and median body weight was 4.2 (3.5‐5.3) kg. Echocardiographic phenotypes identified were (in order of prevalence): obstructive HCM with SAM (20/50), normal (14/50), mitral insufficiency (6/50), non‐obstructive HCM (4/50), dynamic right ventricular outflow tract obstruction (3/50), restrictive cardiomyopathy (2), double‐chambered right ventricle (1/50).

Phonocardiography (performed with concomitant ECG in 21 cats, 7 of which had a HCM‐SAM phenotype) showed among 50 cats, a total of 24 third heart sounds: 20 systolic and 4 diastolic. All of the gallop sounds were S4, and were found in cats with non‐obstructive HCM (1), mitral insufficiency (1), and restrictive cardiomyopathy (2). All obstructive HCM with SAM cats had an additional PCG signal (S1′) after S1 and before S2. It appeared smaller than S1 and variably smaller or higher than S2. It was separated from S1 and occurred between 60 and 80 ms after S1 (Figure 2B).

Agreement between echocardiography and auscultation for observers A, B, and C regarding detection of obstructive HCM with SAM was 0.92, 0.64, and 0.08, respectively (Table 1).

**TABLE 1:** Summarized results of the test of agreement: Kappa value with confidence interval (*K* – 95% CI), and its translation in sensitivity and specificity for each observer.

	Observer A	Observer B	Observer C
*K* (95% CI)	0.92 (0.72 to 0.98)	0.64 (0.38 to 0.8)	0.08 (−0.23 to 0.3)
Sensitivity	1.00	0.90	0.65
Specificity	0.93	0.70	0.43

Observer A heard a third systolic sound in 22 cats, including the 20 cats with obstructive HCM with SAM and in 2 cats without SAM. Both of these 2 cats had a gallop sound on PCG. One cat had restrictive cardiomyopathy and the other mitral regurgitation. Observer A did not hear any third systolic sound in the other 28 cats without obstructive HCM with SAM. Among the 20 cats with obstructive HCM with SAM, observer A graded the third sound as “loud” for 13 cats and “soft” or “intermittent” for 7 cats. Median peak LVOT gradient was significantly higher among the 13 cats with a loud third sound (96; 64‐108 mmHg) compared with the 7 cats with a soft third sound (40; 32‐42 mmHg; *P* < .01).

Observer B detected a third systolic sound in 25 cats, including 18 with obstructive HCM, 3 showing a gallop sound, and 4 healthy cats. Observer C detected a third systolic sound in 30 cats, including 13 with obstructive HCM, 1 with non‐obstructive HCM, 4 showing a gallop sound, 5 showing a systolic murmur, and 7 healthy cats. Population characteristics, echocardiographic phenotypes, and auscultation results are summarized as supplemental information.

Median echocardiographically derived time‐to‐SAM was 68 (63‐72) ms.

The onomatopoeic feature of this sound was described as “P‐Tam‐Ta.” It was best heard over the left cranial sternal border, and sometimes radiated to the right cranial sternal border.

## 4 DISCUSSION

In our study, a third systolic sound was demonstrated by PCG in all cats with obstructive HCM with SAM. The recurrent features of this sound suggest that this finding has a cause‐effect relationship with SAM. Accordingly, the echocardiographic‐derived time‐to‐SAM and the PCG S1‐S1′ interval were consistent. As a consequence, we propose that this additional sound could be called “SAM‐associated sound” (SAM‐AS). A SAM‐AS already has been reported in humans suffering from obstructive HCM, and was estimated to occur in 11% of patients with obstructive HCM.^11^, ^12^, ^13^ The way in which SAM generates a specific sound has been explained by the collision of the mitral leaflet with the interventricular septum or the sudden deceleration of blood flow because of SAM in the LVOT or both. Indeed, the normal heart sounds are not the result of collision of valve leaflets with each other but are produced by the vibration of the myocardium after a mechanical shock.^11^ Gallop sounds are caused by vibration of the myocardium: early diastolic S3 is caused by vigorous left ventricular filling in the presence of volume overload, whereas late diastolic S4 is caused by forced atrial contraction against a stiff ventricle. Based on this concept, is it likely that the anterior mitral leaflet could function as a drumstick and, depending on the force of contraction, could provide a louder or softer SAM‐AS. In cats, third heart sounds are detected very often, more frequently than in dogs. The weight or conformation of the chest or the heart shape, specific to cats, probably plays a role in making these pathological heart sounds more easily audible. Third heart sounds in HCM cats historically have been believed to be and called gallop sounds. The existence of systolic sounds only recently has been hypothesized.^2^, ^5^ As a consequence, a higher clinical relevance has been given to gallop sounds with regard to systolic murmurs because the latter are considered a finding with low sensitivity for detecting HCM in cats.^2^, ^14^ Therefore, detection of a gallop sound remains an important indication for echocardiographic assessment in this species.^5^ However, the veterinary literature lacks a systematic PCG approach to third heart sounds in cats, and until now, it was unknown if they could also occur during systole.^2^ Moreover, for the same reason, the prevalence of systolic vs gallop (diastolic) sounds remains undefined. In our study, the prevalence of SAM‐AS and that of gallop (diastolic) sounds was 40% and 8%, respectively, thus indicating that many third heart sounds actually are systolic rather than diastolic in cats with HCM‐SAM. Of course, a larger number of cats and random recruitment are necessary to confirm this hypothesis. Agreement between detection of SAM‐AS and echocardiography was very good for the cardiologist and good for the trained observer. Various studies in human and veterinary medicine have investigated interobserver variability and the effect of teaching on recognizing cardiac murmurs and gallops.^15^, ^16^, ^17^, ^18^, ^19^, ^20^, ^21^, ^22^ Most of these studies found high interobserver variability,^16^, ^17^, ^18^ and conclude that training has a positive effect on detecting murmurs, especially those of low intensity.^17^, ^19^, ^20^ One of these studies investigated the interobserver variability of 6 veterinarians with different levels of experience in auscultation on recognizing soft systolic murmurs in Cavalier King Charles spaniels caused by mitral valve insufficiency. The degree of agreement was found to be poor.^16^ In a different study, 6 veterinarians with different levels of experience in cardiac auscultation examined 27 Boxer dogs with and without murmurs.^17^ Variable levels of agreement ranging from poor to substantial were found (Cohen's kappa of 0.14‐0.75), with positive correlation depending on the level of experience.^17^ It has been shown in study on humans that even a 1‐hour long online teaching session resulted in significant improvement of auscultation skills.^21^ All of the above‐cited studies were focused not only on the presence or absence of murmurs but also on their quality and intensity. Indeed, murmurs, because of their variable features, are prone to higher subjectivity upon auscultation with respect to a third sound, which does not necessarily warrant a qualitative assessment.^22^ In our study, observer A qualitatively assessed the third systolic sound, but the auscultation agreement compared with echocardiography was tested only for absence vs presence in a binary fashion. This choice was made considering our study as a preliminary step, because it describes a new clinical finding. Agreement regarding the intensity of this third sound would be a next step.

Our study had some limitations. First, PCG tracings were not systematically obtained with simultaneous ECG. Indeed, the rectangular shape and larger dimensions of the stethoscope equipped with ECG made this tool less precise than the other stethoscope without ECG but with classical shape and dimension. Indeed, this latter stethoscope allowed better separation and distinction of SAM‐AS from systolic murmurs in cats with HCM‐SAM, because the systolic murmur was better heard at the left apex whereas the SAM‐AS was better heard at the left cranial sternal border, similar to auscultation using the classical stethoscope. Moreover, to obtain an optimal ECG tracing, the cat's hair should be clipped, but we decided to avoid this practice for the comfort of the cats. Therefore, we were more satisfied with the tracings obtained without ECG, even if not the gold standard, to define the cardiac cycle. The features differentiating S1 from S2 are lower frequency, longer duration, and being the first of 2 grouped sounds (this latter feature obviously not being applicable to high heart rates, as in cats).^7^ When concomitant ECG is lacking, S1 is easily identified on PCG as having a longer duration than S2.^7^ The amplitude is not cited as a criterion, but according to our experience in normal cats with concomitant ECG and examples from the veterinary and human medical literature,^7^, ^8^ we were confident with the assumption that S1 was the tallest PCG signal on the tracings. Therefore, it was simple to identify S1 and S2 (the former being taller and longer than S2) and define the cardiac cycle, just as valve closure aids in defining end‐diastole and end‐systole on echocardiographic images. In particular, S1′ is easily recognized because it is between S1 and S2 which are always present. Another important limitation is that the same observer (observer A) collected data first from auscultation then from echocardiography. These evaluations occurred consecutively on the same cat. Such timing of data collection by observer A may have influenced the echocardiographic result. However, for practical reasons, the study design involved only 1 experienced observer. In this practical context, and because HCM‐SAM is more difficult to diagnose as opposed to the presence of a third sound (which is a new auscultation finding), it was decided to perform auscultation first to prevent the observer A from being influenced by the echocardiographic results. Another limitation, as described above, was the small sample size along with the fact that cats were recruited consecutively and not randomly, and always by the same operator. This design feature could have created a bias regarding the prevalence of the echocardiographic phenotypes as well as of the auscultation findings. Last, all of the factors potentially influencing the intensity of the SAM‐AS and as a consequence the ease of detecting SAM‐AS on auscultation, were not analyzed statistically and thus the fact that in our study loud SAM‐AS was found in cats with higher LVOT gradients cannot be considered to be the rule.

## 5 CONCLUSION

Our study allows for a better understanding of the auscultatory abnormalities in cats with HCM by identifying the relationship between the third systolic sound and obstructive HCM with SAM, and describing its features on PCG. Detection of this sound can be helpful in the diagnosis and management of this heart disease of cats in the preclinical stage, as it could help a trained general practitioner in suspecting obstructive HCM in cats upon auscultation.

## CONFLICT OF INTEREST DECLARATION

Authors declare no conflict of interest.

## OFF‐LABEL ANTIMICROBIAL DECLARATION

Authors declare no off‐label use of antimicrobials.

## INSTITUTIONAL ANIMAL CARE AND USE COMMITTEE (IACUC) OR OTHER APPROVAL DECLARATION

Authors declare no IACUC or other approval was needed.

## HUMAN ETHICS APPROVAL DECLARATION

Authors declare human ethics approval was not needed for this study.

## Supporting information


Summary results.

SAM‐AS auscultation.

## References

[1] Prošek R . Abnormal heart sounds and heart murmurs. In: Ettinger SJ , Feldman EC , Côté E , eds. Textbook of Veterinary Internal Medicine: Diseases of the Dog and the Cat. 8th ed. St. Louis, MO: Elsevier; 2017:220‐221.

[2] Payne JR , Brodbelt DC , Luis FV . Cardiomyopathy prevalence in 780 apparently healthy cats in rehoming centres (the CatScan study). J Vet Cardiol. 2015;17(Supp 1):S244‐S257. 26776583 10.1016/j.jvc.2015.03.008

[3] Fox PR , Keene BW , Lamb K , et al. International collaborative study to assess cardiovascular risk and evaluate long‐term health in cats with preclinical hypertrophic cardiomyopathy and apparently healthy cats: the REVEAL study. J Vet Intern Med. 2018;32:930‐943. 29660848 10.1111/jvim.15122 PMC5980443

[4] Blass KA , Schober KE , Bonagura JD , et al. Clinical evaluation of the 3M Littmann electronic stethoscope model 3200 in 150 cats. J Feline Med Surg. 2013;15:893‐900. 23599254 10.1177/1098612X13485480 PMC11383143

[5] Luis Fuentes V , Abbott J , Chetboul V , et al. ACVIM consensus statement guidelines for the classification, diagnosis, and management of cardiomyopathies in cats. J Vet Intern Med. 2020;34:1062‐1077. 32243654 10.1111/jvim.15745 PMC7255676

[6] Häggström J , Andersson ÅO , Falk T , et al. Effect of body weight on echocardiographic measurements in 19,866 pure‐bred cats with or without heart disease. J Vet Intern Med. 2016;30:1601‐1611. 27573384 10.1111/jvim.14569 PMC5032876

[7] Haggstrom J . In: Kvart C , ed. Cardiac Auscultation and Phonocardiography in Dogs, Horses and Cats. Uppsala, Sweden: TK I Uppsala AB; 2002:14‐106.

[8] El Amine Debbal SM . Analysis of the four heart sounds statistical study and spectro‐temporal characteristics. J Med Eng Technol. 2020;44:396‐410. 32840440 10.1080/03091902.2020.1799095

[9] Donner A , Eliasziw M . A goodness‐of‐fit approach to inference procedures for the kappa statistic: confidence interval construction, significance‐testing and sample size estimation. Stat Med. 1992;11:1511‐1519. 1410963 10.1002/sim.4780111109

[10] Landis JR , Koch GG . The measurement of observer agreement for categorical data. Biometrics. 1977;33:159‐174. 843571

[11] Sakai C , Kawasaki T , Yamano M , Shiraishi H , Kamitani T , Matoba S . Mitral valve systolic anterior motion‐associated sounds in hypertrophic cardiomyopathy. Circ J. 2018;82:1718‐1720. 29070757 10.1253/circj.CJ-17-0714

[12] Rosenblum R , Delman A . Observations on the systolic click and systolic murmur in idiopathic hypertrophic subaortic stenosis. B NY Acad Med. 1966;42:329‐330.

[13] Braunwald E , Lambrew CT , Rockoff SD , et al. Idiopathic hypertrophic subaortic stenosis. I. A description of the disease based upon an analysis of 64 patients. Circulation. 1964;30(Suppl 4):3‐119. 10.1161/01.cir.29.5s4.iv-3 14227306

[14] Paige CF , Abbott J , Elvinger F , et al. Prevalence of cardiomyopathy in apparently healthy cats. J Am Vet Med Assoc. 2009;234:1398‐1403. 19480619 10.2460/javma.234.11.1398

[15] van Staveren MDB , Szatmári V . Detecting and recording cardiac murmurs in clinically healthy puppies in first opinion veterinary practice at the first health check. Acta Vet Scand. 2020;62:1‐8. 32586343 10.1186/s13028-020-00535-1 PMC7315505

[16] Pedersen HD , Häggström J , Falk T , et al. Auscultation in mild mitral regurgitation in dogs: observer variation, effects of physical manoeuvres, and agreement with color Doppler echocardiography and phonocardiography. J Vet Intern Med. 1999;13:56‐64. 10052065

[17] Höglund K , French A , Dukes‐McEwan J , et al. Low intensity heart murmurs in boxer dogs: inter‐observer variation and effects of stress testing. J Small Anim Pract. 2004;45:178‐185. 15116885 10.1111/j.1748-5827.2004.tb00221.x

[18] Germanakis I , Petridou ET , Varlamis G , Matsoukis IL , Papadopoulou‐Legbelou K , Kalmanti M . Skills of primary healthcare physicians in paediatric cardiac auscultation. Acta Paediatr. 2013;102:e74‐e78. 23082851 10.1111/apa.12062

[19] Barrett MJ , Lacey CS , Sekara AE , Linden EA , Gracely EJ . Mastering cardiac murmurs: the power of repetition. Chest. 2004;126:470‐475. 15302733 10.1378/chest.126.2.470

[20] Dhuper S , Vashist S , Shah N , Sokal M . Improvement of cardiac auscultation skills in pediatric residents with training. Clin Pediatr. 2007;46:236‐240. 10.1177/0009922806290028 17416879

[21] Finley JP , Caissie R , Nicol P , Hoyt B . International trial of online auditory training programme distinguishing innocent and pathological murmurs. J Paediatr Child Health. 2015;51:815‐819. 25643859 10.1111/jpc.12839

[22] Lok CE , Morgan CD , Ranganathan N . The accuracy and interobserver agreement in detecting the ‘gallop sounds’ by cardiac auscultation. Chest. 1998;114:1283‐1288. 9824002 10.1378/chest.114.5.1283

