# Supplementary material for: Systolic third sound associated with systolic anterior motion of the mitral valve in cats with obstructive hypertrophic cardiomyopathy
Source: J Vet Intern Med. 2023 Jul 13;37(5):1679–84. doi: 10.1111/jvim.16806 (PMC10472982; doi:10.1111/jvim.16806)
Supplement: Supplementary file 1 — Summary results. [file JVIM-37-1679-s002.pdf]

	Breed	Age (years)	Weight (kg)	Echocardiographic phenotype	Peak LVOT gradient, for cats with obstructive HCM only (mmHg)	Auscultation findings according to Observer A	SAM-AS auscultation Observer A	SAM-AS auscultation Observer B	SAM-AS auscultation Observer C	Agreement Observer A	Agreement Observer B	Agreement Observer C
Cat 1	Domestic shorthair	6,7	4	HCM+SAM	64	SAM-AS LASM 4/6	YES	YES	YES	1	1	1
Cat 2	Domestic shorthair	1,3	5,9	HCM+SAM	41	SAM-AS soft LASM 3/6	YES	YES	NOT	1	1	3
Cat 3	Domestic shorthair	13,3	4,8	H	NA	N	NOT	NOT	YES	2	2	4
Cat 4	Domestic shorthair	0,5	3,2	HCM+SAM	9	SAM-AS intermittent LASM 2/6	YES	YES	YES	1	1	1
Cat 5	Domestic shorthair	7,6	4,5	HCM+SAM	34	SAM-AS soft LASM 3/6	YES	YES	NOT	1	1	3
Cat 6	Domestic shorthair	1,6	6,1	H	NA	N	NOT	NOT	YES	2	2	4
Cat 7	Domestic shorthair	4,6	4,8	H	NA	N	NOT	NOT	YES	2	2	4
Cat 8	Domestic shorthair	13,6	4	HCM+SAM	96	SAM-AS LBSM 3/6	YES	YES	YES	1	1	1
Cat 9	Siamese	15,5	4,2	H	NA	N	NOT	YES	YES	2	4	4
Cat 10	Domestic shorthair	2,2	4	HCM+SAM	71	SAM-AS LASM 3/6	YES	YES	YES	1	1	1

Cat 11	Domestic shorthair	3,1	5,3	HCM+SAM	137	SAM-AS LASM 4/6	YES	YES	YES	1	1	1
Cat 12	Domestic shorthair	1,1	2,7	HCM+SAM	79	SAM-AS LASM 4/6	YES	YES	NOT	1	1	3
Cat 13	Domestic shorthair	7,5	5	H	NA	N	NOT	YES	YES	2	4	4
Cat 14	Domestic shorthair	17,1	3,4	HCM+SAM	30	SAM-AS LASM 3/6	YES	YES	YES	1	1	1
Cat 15	Domestic shorthair	10,6	6,5	HCM+SAM	40	SAM-AS soft LASM 3/6	YES	YES	NOT	1	1	3
Cat 16	Domestic shorthair	1,3	5,6	HCM	NA	N	NOT	NOT	YES	2	2	4
Cat 17	Domestic shorthair	10,8	5,9	HCM	NA	N	NOT	NOT	NOT	2	2	2
Cat 18	Maine Coon	11,0	3,2	HCM	NA	GS	NOT	YES	YES	2	4	4
Cat 19	Domestic shorthair	10,6	5,5	HCM	NA	N	NOT	NOT	NOT	2	2	2
Cat 20	Domestic shorthair	7,9	5,75	H	NA	N	NOT	YES	NOT	2	4	2
Cat 21	Domestic shorthair	0,8	2,5	HCM+SAM	48	SAM-AS soft LASM 2/6	YES	NOT	NOT	1	3	3

Cat 22	Domestic shorthair	0,4	2,54	H	NA	N	NOT	NOT	NOT	2	2	2
Cat 23	Domestic shorthair	0,5	3,75	H	NA	N	NOT	NOT	NOT	2	2	2
Cat 24	Domestic shorthair	0,8	2,8	MR	NA	LASM 2/6+GS	YES	YES	YES	4	4	4
Cat 25	Domestic shorthair	2,3	3	MR	NA	LASM 3/6	NOT	NOT	YES	2	2	4
Cat 26	Domestic shorthair	0,8	4,09	MR	NA	LASM 2/6	NOT	NOT	YES	2	2	4
Cat 27	Domestic shorthair	1,5	5,3	HCM+SAM	108	SAM-AS LBSM 4/6	YES	YES	YES	1	1	1
Cat 28	Domestic shorthair	1,5	3,9	H	NA	N	NOT	NOT	YES	2	2	4
Cat 29	Domestic shorthair	1,7	5,2	HCM+SAM	110	SAM-AS LBSM 4/6	YES	YES	YES	1	1	1
Cat 30	Birman	2,8	4,1	HCM+SAM	108	SAM-AS LASM 4/6	YES	YES	YES	1	1	1
Cat 31	Domestic shorthair	4,7	3	RCM	NA	GS	NOT	NOT	YES	2	2	4
Cat 32	Domestic shorthair	0,8	5,4	RCM	NA	GS	YES	YES	YES	4	4	4

Cat 33	Domestic shorthair	0,9	3,5	DCRV	NA	RBSM 4/6	NOT	NOT	YES	2	2	4
Cat 34	Domestic shorthair	12,3	4,2	HCM+SAM	43	SAM-AS soft	YES	YES	NOT	1	1	3
Cat 35	Domestic shorthair	13,9	3,2	HCM+SAM	30	SAM-AS soft<br>LASM 2/6	YES	YES	NOT	1	1	3
Cat 36	Maine Coon	10,6	9	H	NA	N	NOT	NOT	NOT	2	2	2
Cat 37	Domestic shorthair	16,4	3,25	HCM+SAM	106	SAM-AS	YES	YES	YES	1	1	1
Cat 38	Domestic shorthair	1,6	5,1	HCM+SAM	96	SAM-AS<br>LASM 4/6	YES	YES	YES	1	1	1
Cat 39	Domestic shorthair	12,7	6,2	DRVOTO	NA	RBSM 3/6	NOT	NOT	NOT	2	2	2
Cat 40	Domestic shorthair	0,9	4,2	DRVOTO	NA	SBSM 3/6	NOT	NOT	YES	2	2	4
Cat 41	Domestic shorthair	1,7	4,2	DRVOTO	NA	SBSM 3/6	NOT	NOT	NOT	2	2	2
Cat 42	Domestic shorthair	12,4	5,8	HCM+SAM	64	SAM-AS<br>LASM 3/6	YES	YES	YES	1	1	1
Cat 43	Domestic shorthair	8,7	4,9	HCM+SAM	55	SAM-AS	YES	NOT	YES	1	3	1

Cat 44	Domestic shorthair	5,1	6,2	MR	NA	LASM 2/6	NOT	NOT	YES	2	2	4
Cat 45	Norwegian	0,8	3,2	H	NA	N	NOT	YES	YES	2	4	4
Cat 46	Domestic shorthair	1,1	5,2	MR	NA	LASM 2/6	NOT	NOT	NOT	2	2	2
Cat 47	Persian	11,1	4,1	H	NA	N	NOT	NOT	NOT	2	2	2
Cat 48	Norwegian	12,0	4,3	H	NA	N	NOT	NOT	NOT	2	2	2
Cat 49	Domestic shorthair	1,1	4,25	H	NA	N	NOT	NOT	NOT	2	2	2
Cat 50	Domestic shorthair	6,7	4	MR	NA	LASM 2/6	NOT	NOT	NOT	2	2	2

Table of summary results: Summarized results of the clinical and auscultation findings and scores of agreement (auscultation matched with echocardiography for SAM-AS) in a population of cats screened for heart disease. DCRV: double chambered right ventricle; DRVOTO: dynamic right ventricular outflow tract obstruction; HCM: hypertrophic cardiomyopathy; LASM: left apical systolic murmur; LVOT: left ventricular outflow tract; MR: mitral regurgitation; RCM: restrictive cardiomyopathy; SAM: systolic anterior motion of mitral valve; SAM-AS: SAM associated sound; SBSM: sternal basilar systolic murmur; RBSM: right basilar systolic murmur; GS: gallop sound S4. Scores of agreement test are as follows: 1 = SAM-AS present on auscultation *vs* SAM present on echocardiography; 2 = absent on auscultation *vs* absent on echocardiography; 3 = absent on auscultation *vs* present on echocardiography; 4 = present on auscultation *vs* absent on echocardiography.
